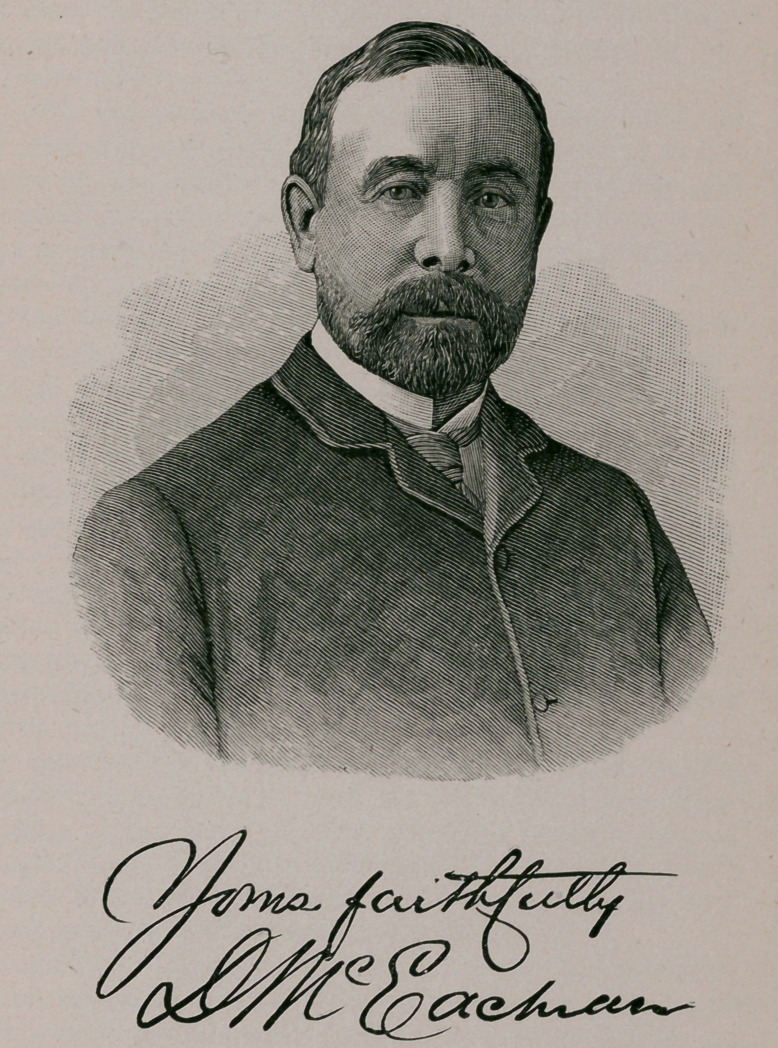# The Montreal Veterinary College, and Its Founder and Principal—Illustrated

**Published:** 1888-01

**Authors:** 


### Art. VII.—THE MONTREAL VETERINARY COLLEGE AND ITS FOUNDER AND PRINCIPAL.

Any account of the origin and history of the college will naturally be preceded by a history of its founder.

Duncan M. McEachran, born in Campbletown, Argyleshire, Scotland, on Oct. 27, 1841, was the son of the late David McEachran, for several years senior bailie of the above town. The family ranks among the oldest in Kintyre ; the Ionic Cross of Campbletown bearing the name of Ed. McEachran, while the tombstones of the family date back to the fourteenth century.

The subject of the present sketch was educated in the Free Church Grammar School of his native place and at the age of seventeen began his professional studies under the distinguished Dick. He graduated as a veterinary surgeon in 1861, became a member of the Royal College of Veterinary Surgeons the same year, and was elected one of the original Fellows of that body on its elevation to the rank of a university in 1875.

In 1862 Mr. McEachran came to Canada and practiced his profession successfully for about three years in Woodstock, Ontario. During this period he gave, during each winter, a course of lectures in Toronto, on professional subjects. This was prior to the establishment of any veterinary school in that Province. He also, during his residence in Woodstock, contributed to the advancement of veterinary medicine in many ways, and especially by lectures given at farmers' meetings, contributions to the agricultural press, and by the publication of a work on veterinary medicine.

In 1866 Mr. McEachran left Ontario to settle in Montreal, not, however, without recognition of his services, for the Board of Agriculture passed a resolution expressing regret at his departure, and he was entertained by a large number of his friends at a public dinner in Woodstock.

Almost immediately on his arrival in Montreal, owing

Yours faithfully

D McEachran to the reputation he had made for himself, powerful friends rallied about him; steps were speedily taken to lay the foundations of veterinary teaching, and in the same year the Montreal Veterinary College was established under the auspices of the late Major Campbell, President of the Board of Agriculture, and of Sir Wm. Dawson, Principal of McGill University.

In 1875 the present sightly and commodious buildings were erected in a central location on Union Avenue, at the expense of the founder and principal, the Government guaranteeing a grant of $1,800 for ten years, with the privilege of sending thirteen French and seven English students for free education in the College.

During his residence in Montreal Professor McEachran has, apart from the duties of his practice and professional teaching, found time to engage in many public undertakings, some of them of the highest importance.

He was for ten years Veterinary Surgeon to the Montreal Field Battery of Artillery.

He has been intimately connected with cattle ranching, Senator Cochrane and himself being the pioneers of that enterprise on a large scale in Canada. In 1881 Principal McEachran visited Alberta, going *via* the Missouri River, driving across the plains from Fort Benton, in Montana, to Morleyville, and publishing a series of letters describing the trip, on his return

He and Senator Cochrane established the ranch bearing the latter's name in 1881, and Mr. McEachran was Vice-President of the same till 1883, when he became general manager of the Walrond Cattle Ranch Company, of which Sir John Walrond is President, and which is now the largest and most successful ranch in the Dominion.

Principal McEachran's efforts to prevent the introduction of foot and mouth disease, pleuro-pneumonia and other infectious diseases from European countries, in which they have proved so destructive, deserves special mention.

In 1875 he urgently pressed upon the government of the Dominion of Canada, the necessity of the establishment of a quarantine system with the object as above stated In April, 1876 he was appointed Chief Inspector, and organized the first Canadian cattle quarantine at Point Levis. He still remains Chief Inspector for the Dominion.

In January, 1879, Professor McEachran was sent by the Canadian government to the United States to investigate the pleuro-pneumonia then prevalent, and visited New York, Long Island, New Jersey, Pennsylvania, Maryland, Virginia and the District of Columbia. The report upon this visit led to important measures being taken by the governments of Great Britain and Canada to prevent diseased animals being imported into these countries, which have since that time, been largely free from the disease. Canada's immunity is directly and almost solely traceable to the action the Government has taken owing to Principal McEachran's representations. The grounds on which he based the action he recommended to the Canadian authorities, can be fully learned from an address delivered at the thirteenth anniversary meeting of the American Veterinary Association, and published in the first number of the *American Veterinary Review.* The appropriateness and force of this appeal needs no comment; but as illustrating the spirit which actuated the writer of that address and the views of the veterinary profession he has always advocated, the concluding paragraph is quoted :

"In conclusion gentlemen, I would suggest to this Association that you should approach the Government and point out the necessity for preventative measures being adopted and urge upon them the importance of recognizing this profession, and ceasing to appoint uneducated men to positions of responsibility, while you have young men educated in science, both able and willing to fill the positions. Let our motto ever continue to be "*Bis Unita Fortior*," and instead of our noble science, for what science next to human medicine can be more noble than that whose object is the relief of suffering in those poor dumb animals which God has given to us to care for, being looked upon as scarcely respectable. We must and will stand side by side with all the other liberal professions, and I certainly do think that if we can bring about the objects of this paper we will do much to deserve the lasting thanks of this great people."

During the entire period of his residence in Montreal Professor McEachran has interested himself in the improvement of the city's sanitary condition. The most recent pathological investigations have demonstrated by new methods, the communicability of infectious and other diseases by milk. This was recognized fifteen years ago by Mr. McEachran, who urged not only the inspection of all milk exposed for sale, but also the regular inspection by experts of all dairy establishments. This fact deserves especial emphasis, for in this at the time referred to and long after he was a voice crying in the wilderness, advocating a view in advance of the times.

A series of articles published in the *American Veterinary Review* of ten years ago, gives ample evidence of the high position he has always claimed for his profession, and his ardent desire that veterinary medical education should be placed upon a higher plane than it then occupied or yet occupies in some institutions. From time to time during the years of his residence in Montreal, Principal McEachran has been in consultation with the practitioners of human medicine with regard to the prevention of the propagation of infectious diseases, the general sanitary condition of the city, etc. The extent to which he has enjoyed the confidence of the general and local governments of the country, and the respect of his fellow-citizens, is evident from the important interests committed to his charge and the general sentiment of the community towards him.

But after all, the most lasting memorial of Principal McEachran's career will probably prove to be the school of veterinary medicine which he founded, and has, with the assistance of his colleagues, maintained for more than twenty-one years in Montreal. The origin of the institution has already been referred to briefly. Principal McEachran began by making the school an embodiment of the principles he has never ceased to advocate from that date to the present time. During the greater part of this period a matriculation examination has been exacted, the requirements for which have been gradually but steadily raised. It is not possible for an illiterate man to enter as a student of the Montreal Veterinary College. The course extends over a period of three years from matriculation, during which an attempt is made to give the student a thorough grounding in all the branches of veterinary education. The College, though not nominally affiliated with McGill University, is practically united with it for teaching purposes ; in fact all the non-professional teaching, except anatomy, is really given by the McGill Professors. This includes courses in Botany, Histology, Physiology, Pathology, Materia Medica and Therapeutics, (there are also special lectures on the veterinary aspects of this branch), and General Pathology. In these various branches the veterinary students are required to attend the same number of lectures and undergo the same examination as the students of human medicine of McGill University. At these examinations some of the highest positions have been attained by veterinary students. The nature of these tests may be learned from the questions on each subject printed in the annual calendars of McGill University Medical Faculty. There are in addition practical examinations to be passed in nearly all of the above subjects and in connection with each of them the opportunity is afforded of doing laboratory work, while in chemistry and physiology this is compulsory. In each of these subjects there are regular demonstrations which all students are required to attend. Provision is made at the Veterinary College for abundant practice in compounding and administering medicines.

The lectures and demonstrations in Pathology in McGill University are supplemented at the Veterinary School by special courses on Entozoa and Cattle Pathology. The students of the College have also the opportunity of attending the autopsies on the human subject at the Montreal General Hospital and learning Virchow's methods of making *post-mortem* examinations.

Turning to professional education, it is at present arranged thus : Principal McEachran lectures on Veterinary Medicine and Surgery ; Professor Baker on Veterinary Anatomy ; Professor C. McEachran on Veterinary Obstetrics and Cattle Pathology. There are other minor courses. The dissecting room, well supplied with subjects, is superintended by Professor Baker, who has assistants.

The College has a large Museum well furnished with models, casts, skeletons, pathological specimens, etc.

On the College premises there is an Infirmary in which sick animals are received throughout the entire year; and as all the principal stables of the city are under the supervision of the Principal and his colleagues, abundant material for clinical and pathological purposes is not wanting. To this must be added the resources of the Quarantine Station, Cattle-yards, Abattoirs, etc.

All the students have the opportunity of joining in clinical practice. Senior students are in turn given charge of cases in the Infirmary under the direction of the professors, and visit the stables in the city accompanied by their teachers to learn the actual practice of their profession. The junior students are appointed assistants to the senior men and aid in dressing and otherwise. As an unusually large number of dogs are kept in Montreal the canine practice of the College clinic is extensive.

Students are required to attend two courses in Cattle Pathology, three in Anatomy, three in Veterinary Medicine and Surgery and two in Obstetrics, while they have the option of taking three courses on any subject of the curriculum. One course of six months is required in Botany, and one of six months in Histology; two courses of six months each in Chemistry and Physiology with practical laboratory work, as before indicated.

Students are invited to attend the Summer Clinics, etc., at the Veterinary College and certain of the summer courses instituted in McGill University for students of human medicine. Many avail themselves of these privileges.

Examinations on the non-professional subjects (except Anatomy) are instituted, as before intimated, by McGill University; the final examinations are conducted by a board appointed by the Council of Agriculture. Last year, among others, two gen lemen, eminent in their profession in the United States, were members of this board. The College grants a diploma and the degree of V. S.

There are two societies in connection with the College: The Veterinary Medical Association, which every student is expected to join, meeting fortnightly, for the reading and discussion of papers, hearing the reports of cases, examination of specimens, etc., and the Society for the Study of Comparative Psychology, which meets monthly for the reading and discussion of papers on animal intelligence. A sketch of the work of this Society since its origin appears in the present number of this JOURNAL.

The College has a library of 140 volumes and the Veterinary Medical Association one of 500 volumes. Most of the journals interesting to the student of comparative medicine are taken regularly. This literature is all freely accessible to the students of the institution.

The school has been conducted on the above described basis* from its commencement, only modifying in the direction of greater breadth and thoroughness to meet the general progress of the age and the special advances of comparative medicine. Sooner than lower its standard the College would close its doors. Nothing shall be said of its graduates, nor or of the quality of the men who give its teaching. Let their work, whether that be practice or teaching, speak for itself. The writer should state that it was with great difficulty that Principal McEachran was induced to consent to the preparation of such an article as this, as one of a proposed series on the various schools; and for this reason as well as the desire to avoid anything that might in the remotest degree savor of "puffing," is it that the present writer has confined himself to a bald statement of facts, with studied abstinence from comment; and though he feels that but scant justice has been done, either to the College or its founder, it seemed the safest course in discharging so delicate a task.

T. W. M.

* It should be mentioned that for many years lectures were delivered in the school in French on the professional subjects by Mons. Daubigny. These were discontinued some four years ago and the education of veterinary students speaking the French language has been since provided for otherwise.

Professor Osler, now of the medical department of the University of Pennsylvania, formerly of McGill University, conducted some of his most important pathological investigations in connection with the Montreal Veterinary College. Mr. Clement, V. S., now of the Pathological department of the United States government, is a recent graduate of the School. As both Dr. Osler and Mr. Clement had a former teaching connection with the Montreal Veterinary College it seems but just to mention them in the present connection.

## Figures and Tables

**Figure f1:**